# MPK6 Kinase Regulates Plasma Membrane H^+^-ATPase Activity in Cold Acclimation

**DOI:** 10.3390/ijms22126338

**Published:** 2021-06-13

**Authors:** Ilian Giordano Ponce-Pineda, Laura Carmona-Salazar, Mariana Saucedo-García, Dora Cano-Ramírez, Francisco Morales-Cedillo, Araceli Peña-Moral, Ángel Arturo Guevara-García, Sobeida Sánchez-Nieto, Marina Gavilanes-Ruíz

**Affiliations:** 1 Departamento de Bioquímica, Facultad de Química, Universidad Nacional Autónoma de México, Mexico City 04510, Mexico; irongado_2405@comunidad.unam.mx (I.G.P.-P.); carmonal@comunidad.unam.mx (L.C.-S.); dlc55@cam.ac.uk (D.C.-R.); fran_0988@hotmail.com (F.M.-C.); apenam0300@hotmail.com (A.P.-M.); sobeida@unam.mx (S.S.-N.); 2 Instituto de Ciencias Agropecuarias, Universidad Autónoma del Estado de Hidalgo, Avenida Universidad Km. 1, Rancho Universitario, Tulancingo-Santiago Tulantepec, Tulancingo, Hidalgo 43600, Mexico; saucedo@uaeh.edu.mx; 3 Department of Plant Sciences, University of Cambridge, Downing Street, Cambridge CB2 3EA, UK; 4 Departamento de Biología Molecular de Plantas, Instituto de Biotecnología, Universidad Nacional Autónoma de México, Cuernavaca, Morelos 62210, Mexico; rturo.guevara@ibt.unam.mx

**Keywords:** cold acclimation, freezing tolerance, H^+^-ATPase, mitogen activated protein kinases, MAPK, MPK3, MPK6, plasma membrane

## Abstract

Cold and freezing stresses severely affect plant growth, development, and survival rate. Some plant species have evolved a process known as cold acclimation, in which plants exposed to temperatures above 0 °C trigger biochemical and physiological changes to survive freezing. During this response, several signaling events are mediated by transducers, such as mitogen activated protein kinase (MAPK) cascades. Plasma membrane H^+^-ATPase is a key enzyme for the plant cell life under regular and stress conditions. Using wild type and *mpk3* and *mpk6* knock out mutants in *Arabidopsis thaliana*, we explored the transcriptional, translational, and 14-3-3 protein regulation of the plasma membrane H^+^-ATPase activity under the acclimation process. The kinetic analysis revealed a differential profiling of the H^+^-ATPase activity depending on the presence or absence of MPK3 or MPK6 under non-acclimated or acclimated conditions. Negative regulation of the plasma membrane H^+^-ATPase activity was found to be exerted by MPK3 in non-acclimated conditions and by MPK6 in acclimated conditions, describing a novel form of regulation of this master ATPase. The MPK6 regulation involved changes in plasma membrane fluidity. Moreover, our results indicated that MPK6 is a critical regulator in the process of cold acclimation that leads to freezing tolerance and further survival.

## 1. Introduction

Plants are organisms that are constantly exposed to different biotic and abiotic stresses. To deal with these situations, plants have developed different mechanisms to quickly perceive changes in their environment and activate rapid and long-term adaptative/defense responses [1,2].

Cold is a major abiotic stress that affects plant growth, development, survival, and geographical distribution of the plants, and it is classified as chilling (temperatures between 0 °C and 15 °C) or freezing stress (temperatures below 0 °C) [3,4]. Throughout evolution, plants from temperate climates have evolved a sophisticated physiological adjustment called cold acclimation, in which freezing tolerance is acquired after gradual exposure to low, but non-freezing temperatures to achieve survival upon freezing conditions [5].

Cold acclimation is a complex process that entails numerous physiological and biochemical changes [6]. Preservation of ionic homeostasis is one of the first and crucial challenges that cells must solve to develop freezing-tolerance. A key enzyme for cell homeostasis is the plasma membrane H^+^-ATPase, a H^+^ pumping protein that establishes a transmembrane H^+^ gradient at the expense of ATP hydrolysis. This gradient regulates the H^+^ concentration in the cytosol and the apoplast, drives the secondary transport of solutes across the plasma membrane, and facilitates cell elongation [7]. Because of its key role in the cell, the activity of the plasma membrane H^+^-ATPase can be regulated through different mechanisms, such as phosphorylation/dephosphorylation events, 14-3-3 protein association, specific lipid interactions, and differential expression of isoenzymes [7,8,9,10,11]. It was discovered that these forms of regulation could be displayed under stress conditions, including low temperatures. For instance, increased levels of gene expression, protein amount, or activity of the plasma membrane H^+^-ATPase were reported after low temperature exposures in fig leaf gourd plants at 6 °C [12], in suspension/cultured sugar beet cells at 0 °C [13], in cucumber roots at 10 °C [14], in camelina and rapeseed plants at 2 °C [15], and in barley seedlings at 5 °C [16]. In contrast, studies with harvested pineapple, a tropical fruit, and cucumber reported a drop in the plasma membrane H^+^-ATPase activity at 10 °C [17,18]. In *Arabidopsis*, it was found that the plasma membrane H^+^-ATPase activity underwent a strong inhibition after 6 h at 4 °C. However, with longer time of cold exposure, the enzyme activity was recovered. Additionally, relative gene expression of *AHA1* and *AHA2* plasma membrane H^+^-ATPase isoforms increased after 12 h at 4 °C, coinciding with a rise in the amounts of plasma membrane H^+^-ATPase and 14-3-3 proteins [19].

Multiple families of kinases and phosphatases are implicated in low temperature responses. For instance, several studies have described that the MAP (mitogen-activated protein) kinases MPK3, MPK4, and MPK6 are activated in response to cold stress in *Arabidopsis* [20,21,22,23,24,25]. These kinases are part of MAPK cascades, which are involved in the magnification and transfer of signals perceived by receptors in order to display specific cellular responses [26,27,28,29,30]. A MAPK module consists in a phosphorylation cascade that is started by a mitogen-activated protein kinase kinase kinase (MPKKK or MAP3K or MEKK), which phosphorylates and activates a mitogen-activated protein kinase kinase (MPKK or MAP2K or MEK) and thereby phosphorylates and activates a mitogen-activated protein kinase (MAPK or MPK). Those enzymes are involved in several physiological processes, such as cellular division, plant growth, and development, as well as in biotic and abiotic responses [30,31,32].

Diverse research lines suggested that the MEKK1-MEK2-MPK4/MPK6 cascade is involved in plant response to low temperatures. CRLK1, a calcium/calmodulin-regulated receptor-like kinase 1, interacts with MEKK1 activating a MAPK cascade at low temperature [33]. Then, MEKK1 phosphorylates MEK2, which in turn phosphorylates and activates MPK4 and MPK6 [21,22]. It has been described that phytosphingosine-1-phosphate (PHS-P) is implicated in the activation of MPK6 in culture cells after one hour to cold exposure [34]. Another study reported that MPK4 is activated downstream PM rigidification, and then MPK4 indirectly activates the *COR15a* gen as a cold response [35].

The mechanism of how *At*MPKs act downstream in the cold response is little understood. Recently, it was reported that MPK3 and MPK6 phosphorylate the transcription factor ICE1, an upstream and positive regulator of the transcription of *CBF* (C-repeat-binding factor) genes that are required for freezing tolerance. The phosphorylation of ICE1, leads to its degradation, negatively affecting the freezing tolerance [24,25]. In contrast, a positive role of MPK6 in freezing tolerance has also been reported. This MPK can phosphorylate MYB15, a transcriptional repressor of cold signaling, diminishing the binding of MYB15 to the *CBF3* promoter [23].

In this study, we describe that *Arabidopsis mpk3* and *mpk6* mutants displayed a plasma membrane H^+^-ATPase activity with different features in terms of kineticsand gene expression upon low temperature treatment. We found that the *mpk6* mutant developed an extremely high freezing sensitivity upon cold acclimation conditions and that its plasma membrane showed an increase in rigidification and in H^+^-ATPase activity. These results indicate that the MPK6 is a critical transducer that positively regulates freezing tolerance in association to a negative regulation of the plasma membrane H^+^-ATPase activity under cold acclimation.

## 2. Results

### 2.1. MPK6 Is Crucial for Freezing Tolerance

**Figure 1:**
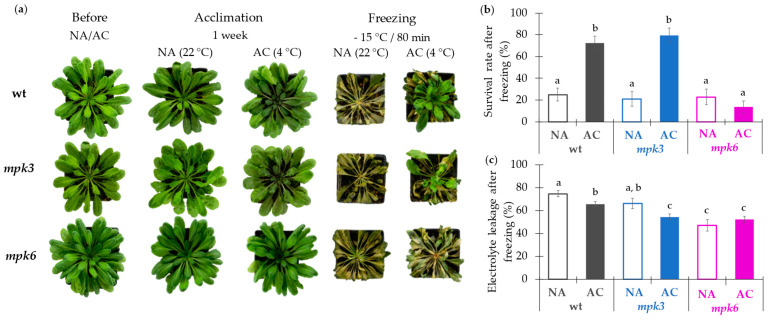
Effect of acclimation and freezing temperatures on the phenotype, survival rate, and electrolyte leakage from wild type, *mpk3*, and *mpk6* plants. Wild type (wt), *mpk3*, and *mpk6* plants were grown at 22 °C for 10 weeks and then subjected to non-acclimation (NA) or acclimation (AC) treatment. For the NA treatment, plants were maintained at 22 °C for another week. For the AC treatment, plants were transferred to a chamber at 4 °C for one week. Then NA and AC plants were shifted to a freezing chamber at −15 °C for 80 min under dark conditions (freezing challenge). For recovery, all plants were moved at 4 °C under dark conditions for 80 min and then to 22 °C for one-week (see Figure S1 for experimental design). (**a**) Acclimation and freezing-tolerant phenotypes. Representative images from NA and AC plants at the end of the acclimation and the recovery periods are shown. Note that plant images are presented at the same scale as denoted for the pot size. (**b**) Survival rate was evaluated at the end of the recovery period. (**c**) Electrolyte leakage was measured immediately after the freezing challenge. Technical details are described under the Materials and Methods section. Representative images are shown from four independent experiments with eight technical replicates each. In (**b**,**c**), data are mean values of eight biological replicates ± SE. α ≤ 0.05 (ANOVA test). Different letters indicate significant differences.

*Arabidopsis thaliana* is a plant that can acclimate to cold temperatures [36]. To explore the effects that the lack of MPK3 or MPK6 kinases can produce in the cold acclimation process, we tested the ability of the *mpk3* and *mpk6* mutants when they were subjected to long-term acclimation and subsequently challenged with a freezing temperature exposure (Figure 1a and Figure S1). Plants from all genotypes looked healthy and fitted at the end of the acclimation period. The plants from all genotypes that were non-acclimated (NA) and directly exposed to freezing were unable of survival. However, upon acclimation treatment, wild type and *mpk3* showed higher survival extent as compared to their respective NA plants exposed to the freezing condition. Even though, their fitness was not equal, as *mpk3* acclimated (AC) plants showed a lower number of green leaves when compared to AC wild type plants. Noteworthy, *mpk6* was extremely sensitive to freezing as reflected by a very low survival just as the NA *mpk6* plants (Figure 1a,b). Altogether, these results implied that MPK6 is a positive regulator of acquired freezing tolerance upon cold acclimation in *Arabidopsis thaliana*.

Electrolyte leakage is generally taken as an indicator of the membrane damage induced by the freezing stress. Thus, we proceeded to measure this parameter in the three genotypes. As shown in Figure 1c, electrolyte leakage in wild type and *mpk3* plants decreased after acclimation process. In contrast, NA *mpk6* plants showed a low leakage level, which remained unchanged upon the AC treatment; those values were even lower than the NA wild type and *mpk3* plants or than AC wild type plants (Figure 1c).

### 2.2. Negative Regulation of the Plasma Membrane H^+^-ATPase Activity Is Exerted by MPK3 in Non-Acclimated Conditions and by MPK6 in Acclimated Conditions

We investigated the mechanism involved in freezing tolerance apparently mediated by MPK6. Since the plasma membrane H^+^-ATPase is a key enzyme affected during multiple stress responses including cold exposure [19], we hypothesized that this enzyme could be one of the molecules associated to the behavior shown by *mpk6* plants. Therefore, our approach was to isolate the plasma membrane vesicles (PMV) from NA and AC *Arabidopsis* leaves from the three genotypes and then to determine the activities and kinetic constants of the H^+^-ATPase.

To evaluate the ultrastructure and purity of the PMV preparations, we performed morphological scanning and immunodetection of membrane marker proteins, such as the aquaporin PIP2-2, the Na^+^/H^+^ antiporter and the sterol methyltransferase 1, localized in the plasma membrane, vacuole, and endoplasmic reticulum, respectively (Figure S2a–e). The results showed high and similar purification level of PVM from the three genotypes, which were enriched in the plasma membrane protein marker and with a low content of vacuolar and endoplasmic reticulum membranes.

**Figure 2:**
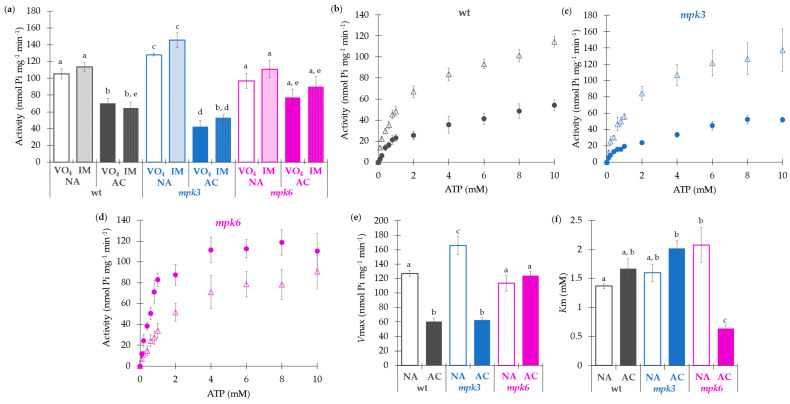
Effect of cold acclimation on the activity and kinetics of the plasma membrane H^+^-ATPase from wild type, *mpk3,* and *mpk6* plants. (**a**) Plasma membrane H^+^-ATPase activity from wild type, *mpk3,* and *mpk6* plants exposed to NA or AC conditions. PMV isolated from NA and AC wild type, *mpk3*, and *mpk6* plants were used to measure ATP hydrolysis in the presence of the specific inhibitor (VO_4_^3−^) or the specific inhibitors (IM; NO_3_^−1^, N_3_^−1^, and MoO_4_^−2^) of some possible contaminant enzymes. ATPase activity was measured at 10 mM of ATP-Mg. (**b**–**d**) Kinetic profiling of the plasma membrane H^+^-ATPase activity from the NA plants (empty circles), or AC plants (solid circles). (**b**) Kinetics of the plasma membrane H^+^-ATPase activity from wild type plants. (**c**) *mpk3* plants. (**d**) *mpk6* plants. (**e**) *V*max values calculated from the panels (**b**–**d**). (**f**) *K*m values calculated from the panels (**b**–**d**). Values of the kinetic constants and Hill numbers are presented in Table S1. Plasma membrane H^+^-ATPase activity was measured from Pi released from ATP. Substrate concentrations were varied between 0.1 to 10 mM ATP-Mg. Values presented in panel (**a**) are the mean values of six biological replicates ± SE. α ≤ 0.05 (ANOVA test). Significant differences are indicated by different letters. Values presented in panels (**b**–**f**) are means of four biological replicates ± SE. α ≤ 0.05 (ANOVA test). Significant differences are indicated by different letters.

The plasma membrane H^+^-ATPase activity was measured as vanadate (VO_4_^3−^)-sensitive ATP hydrolysis, (a specific inhibitor of this enzyme), or as ATP hydrolysis inhibitor mixture-insensitive (IM) including the specific inhibitors NaN_3_, KNO_3_, and Na_2_MoO_4_, which inhibit the mitochondrial ATPase, the vacuolar ATPase, and the acid phosphatase, respectively. These contaminant enzymes could be contributing to the ATP hydrolysis in the assay (Figure 2a). As expected, the values of the ATPase activity were similar in the VO_4_^3−^ and in IM exposure conditions in the PMV from the NA and AC plants from all genotypes.

The H^+^-ATPase activity from wild type plants decreased 30% after the acclimation process (Figure 2a). However, interestingly, lack of function of *MPK3* showed 25% higher activity in comparison to wild type plants. Despite this, the activity from AC *mpk3* plants presented a decrease even more pronounced than the wild type AC plants. The *mpk6* plants again showed different behavior, since H^+^-ATPase from either the NA or AC plants exhibited the same activity, which was coincident with the wild type NA control plants.

### 2.3. Kinetic Characterization of the Plasma Membrane H^+^-ATPase Activity Is Coincident with the Negative Regulatory Roles of MPK3 and MPK6 and Reveals Different Features of the Enzyme

Trying to understand the factors involved in the different plasma membrane H^+^-ATPase activities, we performed a kinetic study of the enzyme activity from the NA and AC plants of the three genotypes (Figure 2b–d). Experimental data described a Michaelis-Menten behavior of the H^+^-ATPase activity from wild type plants, in agreement with previous reports [37], the same that was preserved in the AC condition, and found in the NA and AC *mpk3* and *mpk6* genotypes as well. Accordingly, data showed a good fit to non-cooperative kinetics as an *n* close to 1.0 was obtained with the Hill equation (Table S1). Concerning the catalytic constants, the maximum velocity (*V*max) of the wild type NA plants exhibited a value of 127.2 nmol Pi mg^−1^ min^−1^, which decreased after cold acclimation to 60.5 nmol Pi mg^−1^ min^−1^, matching the activity values previously obtained in Figure 2a (Figure 2b–e and Table S1). *V*max values obtained for NA and AC wild type, *mpk3* and *mpk6*, respectively (Figure 2b–e; Table S1), matched the maximal activity levels shown in Figure 2a.

A significant difference in the affinity for ATP was found in the AC *mpk6* mutant (Figure 2f, Table S1). *K*m from these plants exhibited a low value of 0.64 mM ATP-Mg compared to the *K*m values from the NA wild type and NA *mpk6* plants, which were around 1.37 mM and 2.08 mM ATP-Mg, respectively. The *K*m values from the AC *mpk3* were very similar to the latter, as well. The catalytic efficiency values from the wild type and *mpk3* plants was reduced about three-fold after the acclimation treatment, nevertheless, it was not reduced in *mpk6* plants (Table S1). In fact, it increased 3.5-fold as compared to its NA control. Altogether, these differences in the catalytic constants revealed that the plasma membrane H^+^-ATPase had different functional features depending on the temperature treatment and the presence of MPK3 or MPK6. In addition, the results indicated that MPK3 could be implicated in the negative regulation of the H^+^-ATPase activity in normal conditions of temperature (22 °C), but only MPK6 was involved in the negative regulation of the H^+^-ATPase activity in the response to cold acclimation. Such MPK3 and MPK6 roles were consistent with the determined values of the catalytic constants, which reflected that functional variations of the plasma membrane H^+^-ATPase were induced by the presence of MPK3 or MPK6 in NA or AC conditions.

### 2.4. Contribution of Transcriptional or Translational Effects or Association of Regulatory Proteins to the Negative Control of MPK6 on the Plasma Membrane H^+^-ATPase Activity in Cold Acclimation

**Figure 3:**
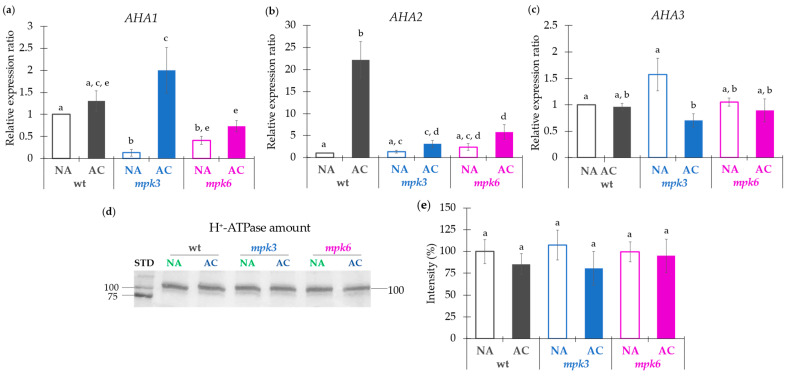
Effect of cold acclimation on the expression of the plasma membrane H^+^-ATPase isogenes AHA1, AHA2, and AHA3 and on the levels of the plasma membrane H^+^-ATPase protein in wild type, *mpk3* and *mpk6* plants. (**a**–**c**) Expression of *AHA1*, *AHA2*, and *AHA3* from NA or AC wild type, *mpk3*, and *mpk6* plants. Plants from the three genotypes were exposed to NA or AC conditions and total RNA was extracted. Relative gene expression of the three isoforms was estimated by qPCR analysis as detailed in Materials and Methods. *TUBULIN2* and *UBIQUITIN4* were used as housekeeping genes. The expression of the genes in untreated wild type was set to 1.0. (**a**–**c**) Levels of plasma membrane H^+^-ATPase protein in PMV isolated from NA or AC wild type, *mpk3*, and *mpk6* plants. (**d**) Immunodetection of the plasma membrane H^+^-ATPase from the wild type, *mpk3*, and *mpk6* plants exposed to NA or AC conditions. (**e**) Densitometry estimation of the intensity of the bands in (**d**); comparison was done between the band intensity from the NA or AC condition respect to the NA wild type band intensity. Loading control gels are presented in Figure S3a. Normalization of the protein bands in the Western blots was performed considering the intensity of the corresponding lane in the Coomassie stained gel and the blot background when different blots were compared. Values presented in panels (**a**–**c**,**e**)are means of six replicates from five independent biological samples ± SE. α ≤ 0.05 (ANOVA test). Different letters indicate significant differences.

In the attempt to explain the causes behind the differences in the profile of the plasma membrane H^+^-ATPase activity, we analyzed the expression levels of *AHA1*, *AHA2,* and *AHA3* genes, which encode three major plasma membrane H^+^-ATPase isoforms expressed in *Arabidopsis* leaves [19,38]. The expression ratios were calculated considering the expression level of every gene from every genotype under NA or AC condition in comparison to that gene expression in the NA wild type (Figure 3).

Control and acclimation conditions induced different *AHA* expression profiles on every genotype (Figure 3a–c). The loss of function of MPK3 or MPK6 suppressed the expression of *AHA1* gene under NA conditions as compared to the NA wild type plants. Upon AC, *AHA1* expression increased 2-fold in the *mpk3* mutant as compared to the NA wild type plants and in a more important way as compared to its own control (NA *mpk3* plants). *AHA2* expression was unaffected in the *mpk3* and *mpk6* mutants under NA. AC increased 22-fold the *AHA2* expression in the wild type and at a very low proportion in the *mpk3* and *mpk6* mutants as compared to the expression in the NA wild type. However, the acclimation was unsuccessful in modifying the *AHA2* expression in the mutants as compared to their NA respective controls. In the case of *AHA3*, its expression was unmodified by the NA conditions in the *mpk3* and *mpk6* mutants as compared to the NA wild type levels. Only a significant decrease was observed in the *mpk3* mutant under AC as compared to its own levels under NA. Thus, no correlation was found between the H^+^-ATPase activity and the transcription levels of the three enzyme isogenes in the three genotypes under AC.

Immunodetection of the plasma membrane H^+^-ATPase in the PMV from all genotypes under NA or AC treatments was performed in order to compare the enzyme levels with the determined H^+^-ATPase activity. A correspondent representative blot is shown (Figure 3d). The comparison here shown was carried out loading PMV samples from NA and AC plants of every genotype in the same gel (Figure S3a). As it was observed in Figure 3d,e, the total amount of H^+^-ATPase was unchanged in all studied conditions and genotypes. Densitometric analysis was performed comparing the H^+^-ATPase levels from all genotypes under non-acclimation and acclimation conditions with respect to the NA wild type plants (Figure 3e). This estimation considered differences in protein loading of the gels and background staining of the independent blots (see Figure S3a). We found no significant differences in the enzyme levels of the AC wild type plants or the NA or AC *mpk3* and *mpk6* plants as compared to the NA wild type plants. This result indicated that the differences in the H^+^-ATPase activity were not associated to the enzyme levels in the PMV from plants of the three genotypes under NA or AC conditions.

The combined results from transcript and protein levels suggested that the differences in the H^+^-ATPase activity determined during cold acclimation must be regulated through other(s) mechanism(s), most probably at a post-translational level, but in which MPK3 and MPK6 are involved.

**Figure 4:**
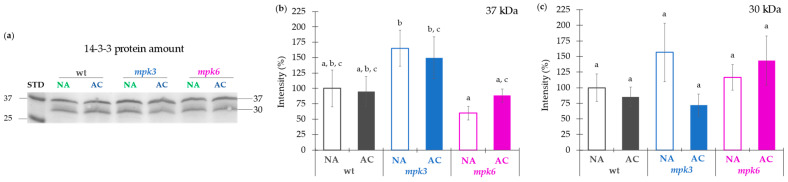
Effect of cold acclimation on the amounts of 14-3-3 proteins from wild type, *mpk3*, and *mpk6* plants. (**a**) Immunodetection of 14-3-3 proteins in PMV isolated from NA or AC wild type, *mpk3*, and *mpk6* plants. (**b**) 37 kDa protein levels and (**c**) 30 kDa protein levels. (**b**,**c**) Densitometric estimation of the intensity of the bands in (**a**); comparison was done between the band intensity from the NA or AC condition with respect to the NA wild type band intensity. Loading control gels are presented in Figure S3a. Normalization of the protein bands in the Western blots was performed considering the intensity of the corresponding lane in the Coomassie stained gel and the blot background when different blots were compared. Values presented in panels (**b**,**c**) are means of six replicates from five independent biological samples ± SE. α ≤ 0.05 (ANOVA test). Different letters indicate significant differences.

It is well established that one activation form of plasma membrane H^+^-ATPase is reliant on the phosphorylation of the penultimate threonine residue at the C-terminus and this is stabilized by the 14-3-3 protein binding to the phosphorylated motif [8]. Therefore, we carried out the immunodetection of 14-3-3 proteins in the PMV from the plants of the three genotypes subjected to NA or AC conditions (Figure 4). The two different bands detected at 37 and 30 kDa could correspond to two from the thirteen 14-3-3 isoforms expressed in *Arabidopsis thaliana* [39]. According to the manufacturer, the antibody used can detect six from those 14-3-3 isoforms.

The 14-3-3 protein levels in the AC condition were estimated using as reference the NA condition for every genotype (Figure 4a). Levels of the 37 kDa protein upon NA and AC conditions were essentially the same in wild type, *mpk3*, and *mpk6* mutants upon NA or AC treatments as observed by the immunoblots and the respective densitometric analysis (Figure 4a–c). Concerning the 30 kDa protein, no changes in content were detected among genotypes, either upon NA or AC treatments. The only difference found was that NA *mpk6* contained lower amount of 37 kDa protein as compared to NA *mpk3*. These results were uncorrelated with the results obtained with the H^+^-ATPase activity described in Figure 2a. Thus, the influence of the membrane environment on the H^+^-ATPase activity was investigated.

### 2.5. Influence of the Plasma Membrane Fluidity on the Negative Control of MPK6 Exerted on the H^+^-ATPase Activity under Cold Acclimation

**Figure 5:**
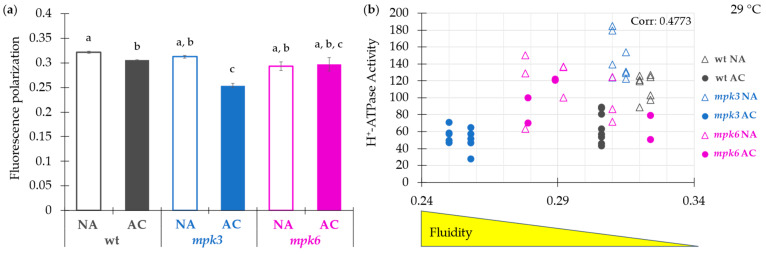
Effect of cold acclimation on the plasma membrane fluidity in wild type, *mpk3*, and *mpk6* plants. Membrane fluidity of was measured by fluorescence polarization (FP) of the fluorescent probe 1-(4-trimethylammoniumphenyl)-6-phenyl-1,3,5-hexatriene *p*-toluenesulfonate (TMA-DPH) at excitation and emission wavelengths, respectively, of 340 nm/417 nm. PMV (300 µg of protein) isolated from *Arabidopsis thaliana* leaves exposed to NA or AC conditions were added to the assay medium and steady-state fluorescence polarization was recorded at 29 °C. Values presented in panel (**a**) are means from 20 to 30 recordings, from two to three independent biological samples ± SE. α ≤ 0.05 (ANOVA test). (**b**) Correlation plot between H^+^-ATPase activity and FP. This was calculated with values from panel (**a**) and Figure 2a using the Pearson model.

It was reported that the H^+^-ATPase from the plasma membrane could be affected by agents that are incorporated in the lipid phase of the bilayer. The mechanism can be explained as a modification of the adjacent milieu environment of the protein or as a perturbation of the organization of the membrane lipid bulk [11]. One way to monitor the state of order of the membrane is through the use of fluorescent probes. Trimethylammonium diphenylhexatriene (TMA-DPH) is a fluorescence polarization probe commonly used to measure the fluidity or ordered sate of the membranes in the region close to the polar heads of the membrane lipids [40]. In order to investigate if membrane fluidity was related to the changes observed in the PM H^+^-ATPase activity in the *mpk3* and *mpk6* mutants under cold acclimation, TMA-DPH fluorescence polarization was determined. A high fluorescence polarization signal corresponds to a low fluidity or high molecular order. It was found that fluorescence polarization decreased in the AC wild type and AC *mpk3*, but was unaffected in the AC *mpk6* PMV (Figure 5a). The analysis to determine a possible association between the H^+^-ATPase activity and fluorescence polarization values, showed a positive correlation between higher activity and higher membrane rigidity (Figure 5b). When we compared this profile with the pattern of the H^+^-ATPase activity in the three genotypes (Figure 2a), there was consistency but in the NA *mpk3* values. These results suggested that acclimation treatment produced an increase in membrane fluidity that was dependent on the presence of MPK6, which in turn, had been found to exert a negative regulation of the H^+^-ATPase activity.

### 2.6. MPK3 and MPK6 Remain Activated after One Week of Cold Acclimation

**Figure 6:**
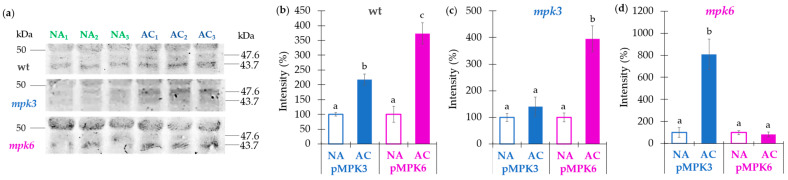
Effect of cold acclimation on the phosphorylation of MPK3 and MPK6 from wild type, *mpk3*, and *mpk6* plants. (**a**) Immunodetection (Western blot) of the phosphorylated forms of MPK3 and MPK6 (pMPK3 and pMPK6, respectively) in NA or AC wild type, *mpk3* and *mpk6* plants. NA_1–3_, and AC_1–3_, correspond to three biological replicates. (**b**–**d**) Densitometric quantification of the protein bands in (**a**). (**b**) Levels of pMPK3 and pMPK6 in NA and AC wild type plants. (**c**) Levels of pMPK3 and pMPK6 in NA and AC *mpk3* plants. (**d**) Levels of pMPK3 and pMPK6 in NA and AC *mpk6* plants. Loading control gels are presented in Figure S3b–d. Normalization of the protein bands in the Western blots was performed considering the intensity of the corresponding lane in the Coomassie stained gel and the blot background when different blots were compared. Values presented in panels (**b**–**d**) are means of three biological replicates ± SE. α ≤ 0.05 (ANOVA test). Different letters indicate significant differences.

Previous studies reported that MPK3 and MPK6 are rapidly activated after cold exposure [21,24,25] and remained active for a short period of time. To know if the active conformation of these MAP kinases persisted during the time used for the acclimation treatment (7 days), we determined MPK3 and MPK6 phosphorylation using an anti-pTEpY antibody, which recognized the active/phosphorylated form of these kinases (Figure 6). According to their respective molecular masses, we found that both enzymes remained activated after one week of cold acclimation in the wild type plants (Figure 6a–d). In the mutants, the expressed reciprocal MAP kinase was detected showing its active conformation (Figure 6a–d).

## 3. Discussion

### 3.1. MPK6 Is a Positive Regulator of the Cold Acclimation Leading to Freezing Tolerance

Cold acclimation is a natural process that prevents lethal injury in plants when they subsequently face freezing temperatures. Therefore, the adjustments that plant cells undergo during the cold, non-freezing temperature exposure involve a diversity of molecular changes that are ultimately translated into freezing tolerance. Cell adjustments are programmed through signaling networks mediated by kinases among other transducer elements. The present study reports the role of the kinase MPK6 as a determinant signaling element in a successful cold acclimation process leading to freezing tolerance in *Arabidopsis*. In addition, the plasma membrane H^+^-ATPase is described here as target of the MPK6 function in the cold acclimation and of the MPK3 in the non-acclimation. This work sheds light on the kinetic features that this master enzyme displays under MPK6 and MPK3 regulation in the regular and low temperatures.

The efficacy of the cold acclimation treatment to the freezing temperatures exposure clearly failed in the *mpk6* mutant, while succeeded in the wild type and *mpk3* mutant plants. The freezing sensitive phenotype has been also reported in the null mutant that lacks MKK2, an upstream activator of MPK6 [21] and in the *Arabidopsis* line that overexpresses the mutation in the phosphorylation site of MYB15, a transcription factor (MYB15^S168A^) downstream of MPK6 during cold response [23]. The latter indirectly confirmed our findings. Although two groups reported that MPK3 and MPK6 are negative regulators in cold acclimation, those results were obtained in different conditions to ours: 10–14 day-old seedlings, different settings of acclimation (3 days), freezing (−5 or −8 °C for 0.5 or 1 h), and recovery (dark and light shifts and 3 days) [24,25]. As it has been demonstrated, MPK6 participates in the control of very important plant developmental programs in *Arabidopsis* [41,42,43]. In addition, it is well established that temperature stress responses are modified accordingly to the intensity, duration, and frequency of the stimulus [44,45,46]. Furthermore, Leuendorf et al. in 2020 [47] demonstrated that cold response in *Arabidopsis* directly depends on age and cold duration treatment. Such circumstances may explain the discrepancies between our results and those from the indicated reports.

### 3.2. MPK6 Influences Membrane Properties Involved in the Freezing Tolerance

The ability of plants to acclimate to cold temperatures is related to the integrity enforcement of cell membranes [48]. In fact, plasma membrane was reported as the first target of low temperature disturbance [35,49,50]. At subzero temperatures, water from the apoplastic space, with a lower solute concentration and ice-nucleation particles, forms ice earlier than in the cell interior; as a consequence, water is lost from the cell to equilibrate solute concentration, producing cell dehydration and membrane damage [51]. Solute leakage has been considered as an experimental proof of the loss of membrane integrity during cold/freezing stress, a concept that was originally developed from processes such as foliar tissues desiccation and dry seeds imbibition [52,53]. However, it was reported that freezing treatment of onion bulbs produced an ion efflux, mainly of K^+^, that was not associated to cell damage [54,55]. Later, a cation leakage through channels activated by reactive oxygen species under stress conditions was described, indicating that not all ion release produced during electrolyte leakage is associated to membrane damage but can be part of signaling pathways [56]. It is possible that the latter effect occurs in the wild type and *mpk3* mutant and not in the *mpk6* mutant, in which a moderate leakage level was observed at high levels of lethality produced by its extremely high freezing sensitivity under control or cold acclimation.

The special behavior of the *mpk6* plasma membrane was observed as well when acclimation treatment was unable to elicit an increase in fluidity in this mutant, as was shown by wild type and *mpk3* plants. Membrane fluidization is necessary for low temperature survival [57]. As previously demonstrated, cold acclimation led to the fluidization of the membrane in wild type plants [58,59]. Furthermore, rigidification of the membrane has been associated to the activation of some MAP kinases [35,60,61]. The impairment in the plasma membrane fluidization of *mpk6* plants could contribute to its extremely low capacity to tolerate freezing temperatures, implying that MPK6 is necessary to promote a fluidity adjustment in the plasma membrane during cold acclimation. Our results suggesting that *mpk6* plasma membranes have lower permeability and plasticity as compared to the wild type and *mpk3* plasma membranes imply that MPK6 can play some role on the regulation of the plasma membrane properties under cold acclimation. The identification and contribution of other membrane components possibly involved in the poor acclimation response shown by the *mpk6* mutant remain to be investigated.

### 3.3. Plasma Membrane H^+^-ATPase Is Negatively Regulated by MPK3 under Non-Acclimation and by MPK6 under Cold Acclimation

We hypothesized that a fundamental enzyme as the plasma membrane H^+^-ATPase could be experienced adjustments as part of the necessary molecular reprogramming to prepare the plant to a possible freezing event, as this is the aim of cold acclimation in nature. It has been reported that the plasma membrane H^+^-ATPase activity increases or decreases upon low temperature exposure depending on the type of plant species or the imposed cold stress conditions [12,13,14,15,16,17]. In *Arabidopsis thaliana*, Muzi et al. (2016) found that activity could decrease or increase as a function of time of cold exposure, being 18 h the longer time they determined. As one of its main findings, the present work described a robust kinetic analysis of the plasma membrane H^+^-ATPase activity that revealed that non-acclimation and cold acclimation, as well as the presence of the MPK3 and MPK6 kinases lead to different functional forms of the enzyme. The cold acclimation treatment moderately diminished the plasma membrane H^+^-ATPase activity from the wild type plants and strongly the one from the *mpk3* mutant. Acclimation was unable to alter the levels of the enzyme activity in the *mpk6* mutant, suggesting that MPK6 was involved in a negative regulation of the plasma membrane H^+^-ATPase activity in response to cold stress. Addressing the possible enzyme features that were behind the activity levels found in the plants from the three genotypes and cold acclimation, the kinetics of their enzyme activities gave some explanations. The affinity of the plasma membrane H^+^-ATPase for its substrate seemed to be in part responsible for the observed activity behavior. AC wild type and *mpk3* mutant showed the lowest plasma membrane H^+^-ATPase activities and the lower affinity for the substrate (*K*m_ATP_ = 1.67 ± 0.42 mM and 2.02 ± 0.32 mM, respectively) and consequently, a reduced catalytic efficiency. In accordance, the higher activity of the plasma membrane H^+^-ATPase showed by the AC *mpk6* mutant presented the major affinity for the substrate (*K*m_ATP_ = 0.64 ± 0.12 mM) and an increased catalytic efficiency. Noteworthy, the *K*m value found for the plasma membrane H^+^-ATPase activity in NA and AC wild type and *mpk3* coincided with the reported *K*m from the isoforms AHA2 [62] or AHA3 [37], whereas the *K*m value for the AC *mpk6* plants was in agreement with that from the isoform AHA1 [37].

### 3.4. The Expression of Plasma Membrane H^+^-ATPase Isogenes Is Regulated by MPK3 and MPK6 and Depends on the AHA Isogene, the MPK, the NA or AC Condition but Is Unrelated to the H^+^-ATPase Activity Extent

The kinetic results suggested a potential MPK6 regulation over the H^+^-ATPase activity through the differential expression of isoforms during cold acclimation. *Arabidopsis thaliana* expresses 11 different isoforms of the plasma membrane H^+^-ATPase, which are preferentially expressed in specific organs or tissues [63]. *AHA1* and *AHA2* genes are abundantly expressed in all organs and tissues [64]. However, some of the isoforms have been described as tissue-specific [65,66,67,68].

While at shorter times of cold acclimation Muzi et al. (2016) found an activated expression of two of the plasma membrane H^+^-ATPase genes in the wild type genotype, our results revealed a different scenery at seven days of cold acclimation. We found that MPK3 and MPK6 displayed a positive non-redundant regulatory activity on *AHA1* expression under basal (NA) condition. Whilst under AC conditions, MPK3 was a negative regulator of *AHA1* expression. *AHA2* expression was independent of MPK3 and MPK6 under NA conditions. However, the expression of *AHA2* required the presence of both, MPK3 and MPK6 under acclimation to reach the maximal level observed in the wild type plants. MPK3 was a positive regulator of *AHA3* expression under AC.

Since the total amount of the plasma membrane H^+^-ATPase protein remained constant in the plants from the three genotypes subjected to the NA or AC conditions, the presence of MPK3 and MPK6 was apparently irrelevant to regulate the amount of the H^+^-ATPase in the plasma membrane. In addition, there was no coincidence between the estimated amount of the enzyme and the H^+^-ATPase activity profiling described in the different genotypes under control and acclimated treatment.

### 3.5. Plasma Membrane H^+^-ATPase Activity Regulated by MPK3 or MPK6 in Non-Acclimation or Cold Acclimation Is Independent on the Amount of Protein Levels or Association of 14-3-3 Proteins

Regarding the comparison of H^+^-ATPase amount in the plasma membrane and the transcription of the AHA isogenes across the different genotypes under NA and AC conditions, the correlation is difficult to attain, since the estimated amounts of H^+^-ATPase performed by Western blot detect total enzyme contents with no isoforms distinction. However, as an example and considering the AC wild type plants, the highest expression of *AHA2* was observed. However, the same AC plants showed a decrease in the H^+^-ATPase activity as compared to the NA condition. Therefore, transcript levels and enzyme activity during cold acclimation seem to be dissociated in the wild type plants at least. Likewise, the H^+^-ATPase activity could be post-translationally regulated by phosphorylation/dephosphorylation of the enzyme or by interaction with other proteins.

The 14-3-3 proteins are the most numerous phosphoprotein interactive proteins in plants, they bind to the H^+^-ATPase as a main target at the plasma membrane, and this interaction activates the enzyme [69,70]. However, our results on the abundance of 14-3-3 proteins from 30 and 37 kDa showed no differences among NA or AC plants of all genotypes. The only decrease was found in the 14-3-3 protein of 37 kDa from the NA *mpk6* mutant with respect to the NA *mpk3* mutant. These values could be related to the low activity observed in the *mpk6* and not in the *mpk3* mutant, since 14-3-3 protein association stabilizes the phosphorylated and active form of the plasma membrane H^+^-ATPase (8).

### 3.6. Plasma Membrane H^+^-ATPase Activity Regulated by MPK6 Is Associated to the Membrane Fluidity

The H^+^-ATPase activity and membrane fluidity results obtained from the NA and AC wild type, *mpk3*, and *mpk6* mutants showed an inverse relationship, suggesting that the plasma membrane H^+^-ATPase requires a rather rigid membrane for a higher ATPase activity. This enzyme is commonly found in membrane nanodomains [71,72,73], which are enriched in sphingolipids and sterols [74]. In order to enhance membrane fluidity during cold acclimation, a decrease of sphingolipids is necessary [75]. Additionally, cold treatment produces a redistribution of the H^+^-ATPase amount in nanodomains, while the total amount of the enzyme remains unchanged in the plasma membrane [76]. This could explain the unvaried levels of the H^+^-ATPase under control and cold acclimated conditions found in the present work, supporting the possibility that the H^+^-ATPase may regulate its activity from its partition to membrane domains with different fluidity and that this is somehow related to the MPK6 signaling. Extrapolating these findings with our results, we could observe that under acclimation conditions fluidity increased and the plasma membrane H^+^-ATPase activity decreased in the wild type and *mpk3* mutant and not in the *mpk6* mutant, which was unable of acquiring membrane fluidity and held a high H^+^-ATPase activity.

Notwithstanding, MPK3 and MPK6 have been recognized with redundant functions, particularly in response to biotic and abiotic stressors. Both kinases were reported activated upon cold acclimation in *Arabidopsis thaliana* [77,78]. Discerning the specific contribution of the absence and presence of the two MPKs in one single mutant is difficult to achieve, as the double mutant *mpk3 mpk6* is non-viable [24]. Using several lines of biochemical evidence and a reverse genetics approach, the present study reveals that MPK6, but not MPK3, acts as a critical inducer of freezing tolerance. This mode of action of MPK6 is associated to a role of negative regulator of the plasma membrane H^+^-ATPase activity. Further investigation is required to describe the details of the negative regulation of the plasma membrane H^+^-ATPase activity by MPK6 in cold acclimation. Very few kinases of the plasma membrane H^+^-ATPase have been described [79,80,81], and no previous reports have disclosed the regulation of the plasma membrane H^+^-ATPase by a MAP kinase cascade.

## 4. Materials and Methods

### 4.1. Plant Material and Growth Conditions

*Arabidopsis thaliana* ecotype Columbia-0 (Col-0), *mpk3* (RNAi silenced) and *mpk6-2* (SALK_073907) were used in this study. *Arabidopsis* seeds were germinated and grown at 22 ± 2 °C under 8/16 h light/dark photoperiod in pots that contains growing mixture containing 3 parts of Mix 4 Aggregate plus (Sunshine, Sun Gro Horticulture; VBC, Canada Ltd.), 1 part of Premium Vermiculite (Sunshine, Sun Gro Horticulture; VBC, Canada Ltd.), and 1 part of Dica Mex Agrolite (Dicalite de México S.A. de C.V.; Tlalnepantla, Mexico State) with adequate humidity for four weeks. Then one or three seedlings were transplanted to independent pots with the same growing mixture. Pots were placed on trays in a greenhouse, covered with translucid domes, and maintained with natural photoperiod at 22 ± 2 °C. Plants were watered with Hoagland II solution [82] or water in alternate days.

### 4.2. Physiological Analysis

Acclimation treatment. Adult plants were transferred to a growing chamber at 4 °C (cold acclimated, AC) or maintained at 22 °C (non-acclimated, NA) for one week (Figure S1). After that, some plants were used for the freezing challenge to assess their freezing tolerance. The rest of the plants were used to harvest the leaves, which were frozen and stored at −72 °C for later analyses.

Freezing challenge. This was performed by exposure of one-week NA or AC plants to −18 °C for 80 min in dark (Figure S1). Then, plants were tested for ion leakage or to assess survival rate and fitness capacity. For the two latter estimates, NA or AC challenged plants were transferred to a 22 °C growth chamber under an 8/16 h light/dark photoperiod; after 7 days (recovery period), plant survival was recorded.

Electrolyte leakage assay. Seven medium size leaves from NA or AC challenged plants were cut and put on a flask with 80 mL of distilled water under constant stirring and maintained at 4 °C for 40 min as described in [83].

### 4.3. Subcellular Fractionation

Cytosolic fraction was obtained from NA and AC plants, which leaves were frozen and homogenized in buffer containing 250 mM sorbitol, 50 mM HEPES/BTP pH 7.8, 10 mM NaF, 5 mM dithioerythritol, 1 mM EDTA, 1 mM KCl, 1 mM Na_3_VO_4_, 1 mM PMSF, and protease inhibitor cocktail [84] using a Tissue-Tearor (Thomas-Scientific) for 3 min and centrifuged at 12,000× *g* 20 min at 4 °C. The crude extracts were frozen and stored at −72 °C.

PMV were isolated from microsomal fractions obtained from leaves of NA and AC plants as detailed in [84]. The PM were purified by aqueous two-phase partitioning systems with polyethylene glycol 3350/dextran T-500 as polymers.

### 4.4. ATPase Activity and Kinetic Assays

Activities and kinetic analysis from plasma membrane H^+^-ATPase were performed as previously described [85]. ATP hydrolysis was measured by incubation of the PMV in a reaction medium that included Na3VO4, a specific inhibitor of the plasma membrane H^+^-ATPase or a mixture of NaN_3_, KNO_3_, and NaMoO_4_, specific inhibitors of mitochondrial ATPase, tonoplast ATPase, and acid phosphatase, respectively. They can be present as minor contaminants that hydrolyze ATP. ATP hydrolysis was determined as Pi release using a colorimetric method [86].

### 4.5. Plasma Membrane Fluidity Assay

This was measured by fluorescence polarization (FP) of the fluorescent probe 1-(4-trimethylammoniumphenyl)-6-phenyl-1,3,5-hexatriene *p*-toluenesulfonate (TMA-DPH) at excitation and emission wavelengths, respectively, of 340/417 nm. The probe was dissolved in absolute dimethylformamide to a final concentration of 1 mM. PMV (300 µg of protein) isolated from *Arabidopsis thaliana* leaves exposed to NA or AC conditions were added to a solution of 620 mM sorbitol, 5 mM KH_2_PO_4_ (pH 7.8), 0.1 mM EDTA, and 1 µM TMA-DPH up to a final volume of 2.0 mL. The sample was then incubated under agitation at 1 °C for 15 min before measuring fluidity. Steady-state fluorescence polarization was recorded at 29 °C. Steady-state FP values were determined in a SLM-AMINCO 48000 spectrofluorometer (SLM Instruments Inc., Urbana, IL, USA) equipped with light polarizers.

### 4.6. RNA Extraction and qPCR Analysis

RNAs were extracted from leaves of NA and AC adult plants using TRIzol reagent (Invitrogen, Carlsbad, CA, USA) following the manufacturer’s instructions. Total RNA was proved and quantified on a NANODROP 2000 (Thermo Scientific Inc. Waltham, MA, USA). RNA with absorbance ratios close to 2.0 at 260 and 280 nm were selected [87]. RNA integrity was evaluated on a 2% agarose gel. The first-strand cDNA was synthesized from 1 µg RNA using oligo-dT and ImProm-IITM Reverse Transcription System (Promega, Madison, WI, USA) and stored at −20 °C until use.

The 10× diluted cDNA samples were used as templates and quantitative real-time PCR was performed by using SYBR Green Master Mix (Applied Biosystems) with gene-specific primers (Table S2) on a 7500 real time PCR system (Life Technologies). The β-tubulin and Ubiquitin 4 genes were used as the housekeeping standard [88]. The transcript ratio was calculated using the model developed by Pfaffl [89] and modified by Hellemans et al. [90]:

$$\mathrm{Ratio}\;\mathrm{transcripts}=\frac{E_{\mathrm{target}}^{{\Delta \mathrm{CP}}_{\mathrm{target}\left(\mathrm{control}-\mathrm{sample}\right)}}}{\sqrt[{f}]{\prod_{0}^{f}E_{{\mathrm{ref}}_{0}}^{{\Delta \mathrm{CP}}_{{\mathrm{ref}}_{0}\left(\mathrm{control}-\mathrm{sample}\right)}}}}$$

where E_target_ is the amplification efficiency of the target gene, ΔCP_target_ is the Ct difference between control and treated samples, E_ref_ is the amplification efficiency of the housekeeping gene, and ΔCP_ref_ is the housekeeping Ct difference between control and treated samples.

### 4.7. Protein Determination

Protein content in the membrane preparations were determined according to the Lowry procedure modified by Peterson [91] using BSA as standard. Protein concentration in cytosolic fractions was estimated with the Bradford protein assay kit (Bio-Rad, Hercules, CA, USA) using BSA as standard.

### 4.8. Immunoblotting

Proteins were separated by SDS-PAGE (10% acrylamide gel) using the technique from Schägger and von Jagow [92]. Equal amounts of total protein were used in all protein analyses performed, the same loading amount was verified running parallel gels used for blotting and Coomassie Blue (Sigma-Aldrich Corp. St. Louis, MO) staining. The gel was transferred onto PVDF membranes (Immobilon-P, Millipore Corp. Bedford, MA) by electro-transfer at 22 V for 2.5 h The membrane was enhanced with Western Blot Signal Enhancer (Pierce^®^, Thermo Scientific, IL, USA) and blocked in TBST buffer containing 2% free fat milk powder and further incubated with primary antibody and second antibody. Finally, the bands were detected using alkaline phosphatase reaction (1:2500, Millipore). Antibodies used for immunoblotting were as follows: anti PIP2;1, PIP2;2, PIP2;3 (1:1000, Agrisera AS09 491), anti-Na^+^/H^+^ exchanger 1 (1:1000, Agrisera AS09 484), anti-SMT1 (1:1000, Agrisera AS07 266), anti-H^+^-ATPase (1:10,000, Agrisera AS07 260), anti-14-3-3 proteins (1:1000, Agrisera), and anti-Phospho-p44/p42 MAPK (anti-pTEpY) (1:2000, Cell Signaling Technology).

### 4.9. Electron Microscopy

Membrane pellets were fixed with glutaraldehyde (3%, *v*/*v*) in 100 mM phosphate buffer (pH 7.0, 2 h, 4 °C) and osmium tetroxide (1% [*v*/*v*], buffered at pH 7.0, 2 h, 4 °C). The samples were dehydrated embedded and cut in ultrathin slices as described in [84]. Sections were observed with a JEOL 1200 EXII electron microscope operated at 60 kV. Images were processed with Photoshop imaging software (version 8.0.1, Adobe Systems, http://www.adobe.com/products/photoshop/family, accessed on 10 June 2021). Assembly of the figure panels was performed with ImageJ (version 1.44p) and Gimp (version 2.4.0) programs.

## Supplementary Materials

The following are available online at https://www.mdpi.com/article/10.3390/ijms22126338/s1.
