# Supplementary material for: MPK6 Kinase Regulates Plasma Membrane H+-ATPase Activity in Cold Acclimation
Source: Int J Mol Sci. 2021 Jun 13;22(12):6338. doi: 10.3390/ijms22126338 (PMC8232009; doi:10.3390/ijms22126338)
Supplement: Supplementary file 1 [file ijms-22-06338-s001.zip › Supp Fig S1-S3.pdf]

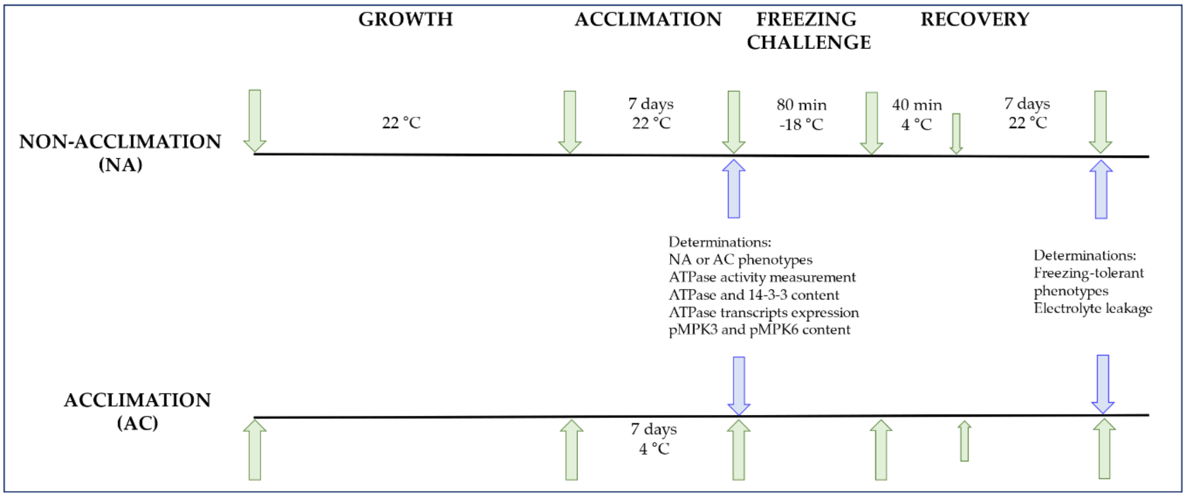

Figure S1. Experimental design followed to study the roles of MPK3/6 and the plasma membrane H^+-ATPase in cold acclimation and freezing tolerance. *Arabidopsis thaliana* plants from wild type and *mpk3* and *mpk6* mutants were grown during 10 weeks at 22 °C and then they were subjected to acclimation conditions (AC) or to control, non-acclimation conditions (NA). When acclimation period just finished, the experimental measurements shown were carried out. Other plant groups continued with the freezing challenge treatment and the recovery time and then their phenotypes were photographically recorded. Temperature and time exposures for every treatment are indicated in the time line.

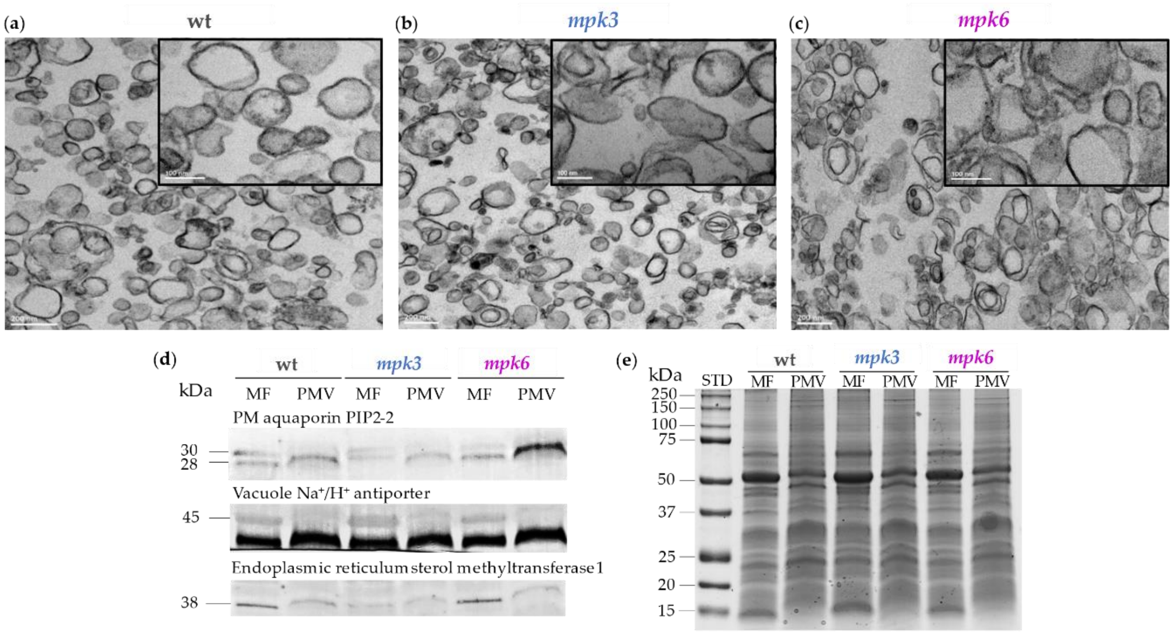

Figure S2. Assessment of PMV purification from wild type, *mpk3* and *mpk6* plants. Plasma membrane vesicles (PMV) were isolated from *Arabidopsis thaliana* leaves by the two-phase partitioning procedure as described under Materials and Methods. (a)–(c) Ultrastructure of PMV preparations. Transmission electron micrographs from PMV obtained from wild type (**a**), *mpk3* (**b**) and *mpk6* (**c**) plants (bar= 200 nm). Insets shown magnifications (bar= 100 nm). (**d**) Presence of enzyme marker enzymes in crude membrane fractions (microsomal fraction, MF) and purified PMV. Membrane proteins were separated by SDS-PAGE and immunodetected (western blot). The membrane markers were: aquaporin PIP2-2 to plasma membrane, $Na^{+/}H^{+}$ antiporter to vacuolar membrane and sterol methyltransferase 1 to endoplasmic reticulum. (**e**) A replicate gel stained with Coomassie blue is showed as loading control. All images shown are representative from at least three biological replicates.

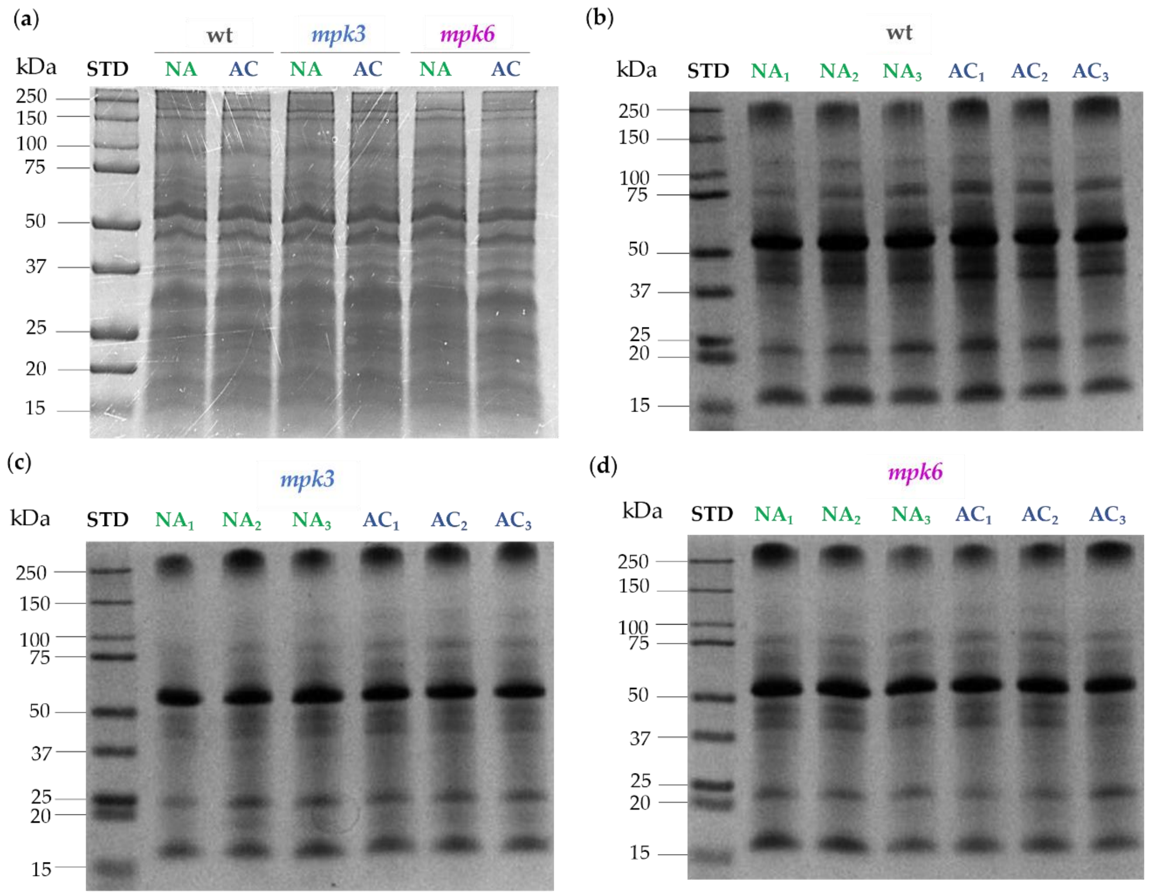

Figure S3. Replicate gels stained with Coomassie blue as protein loading controls from the immunoblots shown in Figures 3-5. (**a**) Gel corresponding to the three technical replicates performed for blotting the wt, *mpk3* and *mpk6* PMV proteins and decorated with the plasma membrane H^+-ATPase antibody (Figure 3) or decorated with the 14-3-3 antibody (Figure 4). (**b-d**) Gels corresponding to the three technical replicates performed for blotting the wt, *mpk3* and *mpk6* proteins from the cytosolic fractions and decorated with the pTEpY antibody (Figure 6). NA_{1-3} and AC_{1-3} correspond to three biological replicates from the wild type, *mpk3* and *mpk6* plants exposed to NA or AC conditions.
