# Supplementary material for: MPK6 Kinase Regulates Plasma Membrane H+-ATPase Activity in Cold Acclimation
Source: Int J Mol Sci. 2021 Jun 13;22(12):6338. doi: 10.3390/ijms22126338 (PMC8232009; doi:10.3390/ijms22126338)
Supplement: Supplementary file 1 [file ijms-22-06338-s001.zip › Supp Tables 1,2.pdf]

Table S1. Kinetic parameters from the plasma membrane H^+-ATPase from NA and AC wild type, *mpk3* and *mpk6* mutants. PMV isolated from NA and AC wild type, *mpk3* and *mpk6* plants were used to measure plasma membrane H^+-ATPase activity as Pi released from ATP. Substrate concentrations were varied between 0.1 to 10 mM ATP-Mg. Kinetic treatment of data with Michaelis–Menten and Hill equations was performed. Experimental curves are shown in Figure 2 (b-d). Values are means of four biological replicates ± SE.

Kinetic parameter		wt		*mpk3*		*mpk6*	
		**NA**	**AC**	**NA**	**AC**	**NA**	**AC**
Michaelis-Menten	*V*max (nmol Pi mg^{-1} min^{-1})	127.20 ± 4.06	60.49 ± 4.81	165.80 ± 12.29	62.74 ± 3.38	113.32 ± 11.25	124.29 ± 6.12
	*K*m (mM)	1.37 ± 0.14	1.67 ± 0.42	1.60 ± 0.37	2.02 ± 0.32	2.08 ± 0.60	0.64 ± 0.12
	*V*max/*K*m (s^{-1})	1547.44 ± 483	603.69 ± 191	1727.08 ± 554	517.66 ± 176	908.01 ± 312	3236.72 ± 850
Hill	*V*max (nmol Pi mg^{-1} min^{-1})	114.64 ± 9.22	55.73 ± 13.25	137.81 ± 8.62	53.61 ± 9.85	96.55 ± 8.51	122.49 ± 4.91
	K′ (mM)	1.24 ± 0.27	1.61 ± 1.07	1.22 ± 0.21	1.61 ± 0.75	1.46 ± 0.35	0.72 ± 0.08
	n	1.07 ± 0.17	0.97 ± 0.36	1.11 ± 0.15	1.19 ± 0.41	1.24 ± 0.24	1.24 ± 0.16

Table S2. qPCR primers used for amplification and quantitative determination of transcripts from the AHA 1-3 plasma membrane H^+-ATPase isoforms. PCR analysis was performed as described under Materials and Methods section.

Gene	Accessory number	Oligo name	Sequence (5′-3′)	Product size
AHA1	AT2G18960	qAHA1-FW	TCTCAGCGTTCATGGTTCTG	222
AHA1	AT2G18960	qAHA1-RV	ACCTCACACCGAACTTGTCC	
AHA2	AT4G30190	qAHA2-FW	TGCTCAAAGGACACTTCACG	173
AHA2	AT4G30190	qAHA2-RV	GCTTCACGACTGATTCCACA	
AHA3	AT5G57350	qAHA3-FW	GAGGAGAGGGAAGCACAATG	155
AHA3	AT5G57350	qAHA3-RV	CTAAGCCTTGCGATCTCAGC	
TUB2	AT5G62690	qTUB2-FW	GTTCTCAGCAGTACCGTTCC	178
TUB2	AT5G62690	qTUB2-RV	CTCCACAAAGTAGGACGAGTTC	
UBQ4	AT5G20620	qUBQ4-FW	CAACCCTTCATTTGGTGCTT	162
UBQ4	AT5G20620	qUBQ4-RV	GTCTCTGCTGATCTGGTGGA	
